# Characterisation of human astrovirus in a diarrhoea outbreak using nanopore and Sanger sequencing protocols

**DOI:** 10.1186/s12985-023-02224-7

**Published:** 2023-11-14

**Authors:** Jinhui Li, Lang Yang, Kaiying Wang, Zhiyong Gao, Peihan Li, Yanfeng Lin, Leili Jia, Quanyi Wang, Hongbin Song, Peng Li

**Affiliations:** 1 Chinese PLA Center for Disease Control and Prevention, Beijing, China; 2 Huadong Research Institute for Medicine and Biotechniques, Nanjing, China; 3 Beijing Center for Disease Prevention and Control, Beijing, China

**Keywords:** Diarrhea outbreak, Human astroviruses, Nanopore sequencing, Sanger sequencing, Whole genome

## Abstract

Human astroviruses (HAstV) are etiologic agents of acute gastroenteritis that most often afflict young children and elderly adults. Most studies of HAstV have focused on epidemiology. In this study, we collected 10 stool samples from a diarrhea outbreak from a diarrhea sentinel surveillance hospital in Beijing. Samples were evaluated immediately using parallel multiplex RT-qPCR and nanopore sequencing, and were then amplified by designed primers and Sanger sequencing to obtain whole genome sequences. Six isolates were categorized as HAstV-5 and subjected to whole genome analysis to characterize their genetic variation and evolution. Full genome analysis revealed low genetic variation (99.38–100% identity) among isolates. Phylogenetic analysis showed that all isolates were closely related to domestic strains Yu/1-CHN and 2013/Fuzhou/85. The recombination breakpoint of the six isolates was located at 2741 bp in the overlap region of ORF1a and ORF1b, similar to those of Yu/1-CHN and 2013/Fuzhou/85. Overall, our study highlights the combined use of RT-qPCR and sequencing as an important tool in rapid diagnosis and acquisition of whole genome sequences of HAstV.

**Supplementary Information:**

The online version contains supplementary material available at 10.1186/s12985-023-02224-7.

## Highlights

We parallel used multiplex RT-PCR and nanopore sequencing to identify Human astroviruse from 10 stool samples of a diarrhea outbreak from a diarrhea sentinel surveillance hospital in this study. Six isolates were categorized as HAstV-5 and subjected to whole genome analysis to characterize their genetic variation and evolution.It is showed that the combined use of RT-PCR and sequencing as an important tool in rapid diagnosis and acquisition of whole genome sequences of HAstV.

**Supplementary Information:**

The online version contains supplementary material available at 10.1186/s12985-023-02224-7.

## Introduction

Human astroviruses (HAstV) are small positive-sense single-stranded RNA viruses. Since their discovery in 1975, they have been recognized among the most prevalent pathogens causing acute infantile gastroenteritis worldwide [1]. Symptoms and signs of HAstV infections last 1 to 4 days, and feature watery diarrhea that can be less commonly accompanied by fever, headaches, abdominal pain, and anorexia. However, many infections in healthy children and adults tend to be asymptomatic [2, 3]. Notably, HAstV, especially HAstV-4 and HAstV-8, may cause extra-gastrointestinal symptoms in immunocompetent individuals. However, as there are currently no diagnostic tools capable of detecting all 16 known HAstV species, their prevalence is almost always underestimated [4]. Improved molecular techniques for detection, diagnosis, and surveillance are essential to establish the clinical importance of HAstV and to determine their prevalence more accurately [5].

The HAstV genome has a length of 6.2–7.9 kb and contains three open reading frames (ORFs): ORF1a, ORF1b and ORF2. ORF1a and ORF1b encode nonstructural proteins involved in RNA transcription and replication, while ORF2 encodes structural proteins and is commonly used for genotyping [6]. A 348-bp segment located between nucleotides 258 and 606 of full-length ORF2 has been used frequently for genotyping. Eight serotypes of HAstV have been identified, including recently discovered novel HAstV-MLB and HAstV-VA strains; however, HAstV-1 remains the most prevalent strain worldwide [7–9]. As viruses with RNA genomes, nucleotide mutations and recombination events are, among other factors, important in their genome evolution [10]. Recombination frequency depends on the degree of similarity between the involved sequences, length of viral genome, and the presence of recombination hot spots [11].

Recent epidemiologic data on HAstV-induced gastroenteritis in China are limited. Most published studies have focused primarily on HAstV infections in children, especially those under 5 years of age [12]. In most studies, RT-qPCR was used for nucleotide sequence amplification to identify genotypes HAstV1-8, while whole genome sequencing was used less commonly.

In this study, we combined quantitative RT-PCR (RT-qPCR) with MinION and Sanger sequencing for diagnosis and characterization of HAstV in a diarrhea outbreak to enable the discovery of novel strains and to improve our knowledge of HAstV circulation.

## Materials and methods

A total of 10 fecal samples were collected during an acute gastroenteritis outbreak in March 2019 in a diarrhea sentinel monitoring hospital in Beijing. Fecal samples (0.1 g for solid or 100 μl for liquid) were suspended in 1mL phosphate buffered saline (PBS) to prepare an approximately 10% solution. This solution was vortexed at least 3 times, then allowed to stand for 10 min, and then centrifuged at 8,000 ×*g* for 5 min.

The Viral RNA was extracted from 200 μL to 10% fecal suspension of each sample with the QIAamp® MinElute Virus Spin Kit (QIAGEN, 57,704) following the manufacturer’s instruction. RT-qPCR specific assays were performed using a 24-diarrheal pathogen nucleic acid detection kit (A + B pre-made plate/fluorescent PCR) (BioGerm, YZ-FX-901) according to the manufacturer’s instruction on a Stratagene Mx3000P (Thermofischer, Waltham, MA, USA) and a CFX96 Touch™ Real-Time PCR Detection System (Bio-Rad, Hercules, CA, USA). The kit could screen 24-diarrheal pathogen including norovirus I and II, rotavirus A, rotavirus B, rotavirus C, enteric adenovirus, human astrovirus, Sapporo virus, *Campylobacter*, *Vibrio parahaemolyticus*, *Listeria monocytogenes*, *Aeromonas hydrophila*, *Vibrio cholerae*, *Bacillus cereus*, *Yersinia pseudotuberculosis*, *Salmonella*, *Escherichia coli*, *Campylobacter jejuni*, *Vibrio fluvialis*, *Staphylococcus aureus*, *Vibrio mimicus*, *Yersinia enterocolitica*, *Shigella* and *Plesiomonas shigelloides*. All 10 samples were screened for the above 24 pathogens by RT-qPCR.

Extracted RNA was subjected to the synthesis of cDNA using NEBNext Ultra II RNA First Strand Synthesis Module (New England Biolabs, USA) and NEBNext Ultra II Non-Directional RNA Second Strand Synthesis Module (New England Biolabs, USA). cDNA aliquots (10 μL) of 10 samples were mixed and enriched with AMPure XP beads (Beckman Coulter, A63881) by 1.8 × volume content. A 15-μL enriched cDNA was used for library preparation. For the MinION (Oxford Nanopore Technologies), we used the Rapid Barcoding Kit (SQK-RBK004) for library preparation and R9.4.1 flow cell (FLO-MIN106) for sequencing according to the manufacturer’s protocol. Sequencing run was maintained for 24 h with the fast base calling mode. Adapters were trimmed and reads with quality scores below 8 were filtered using MinKNOW (v23.04.6).

Based on the results of RT-qPCR and nanopore sequencing, the full length of the HAstV genome of six positive samples needed to be further amplified and subjected to Sanger sequencing. cDNA was synthesized using FastKing RT kit (Tiangen, KR116) following the manufacturer’s instructions, and was then amplified by primers of AstVp1 ~ AstVp12 and random primer set of TX30SXN/DM4 with the 2×Taq Plus PCR Mix (Tiangen, KT205) [13, 14]. The PCR conditions were set as follows: 94℃ held for 3 min, following by 35 cycles of 94℃ for 30s, 40℃ for 30s, 72℃ for 1 min, and final extension at 72℃ for 7 min.

For regions of HAstV genomes that were not obtained by whole genome and nanopore sequencing, we redesigned 4 pairs of primers to close the gap (Table 1). All PCR products were subjected to Sanger sequencing (Shenggong, Shanghai, China).

**Table 1:** Primers used to confirm the isolates sequence

Primer name	Objective	Sequence (5′ to 3′)
AstVp1	To amplify the full length of the HAstV genome	CCAARAGGGGGGTGGYGATTGGC
AstVp2		TYCCATTRRCRTCACGGATYTC
AstVp3		GMACRACCACGTCATTRTTTGY
AstVp4		TCAAATTCYACATCRTCACCAAC
AstVp5		TGGYTAYCCTGAYTATGATGATG
AstVp6		YACTATYTGCCGRATRTCAGAAT
AstVp7		GAAKCAYATGGDTGGGCACCAT
AstVp8		TGACAATKTTACGGACACGTTG
AstVp9		GACCAAAGAAGTGATGGCTAGC
AstVp10		TAGGYTGRTTCATYTGKGTRAAYT
AstVp11		GYTAYCARGATGCHYTRTCYAAT
AstVp12		CTGATTAAATCAATTTTAAATG
Ast1F	To close the gap of nanopore sequencing	TTGGAGAAAGGTCTGGATCG
Ast1R		GAAGGGGTTGGTACGGATTT
Ast2F		GAGCCAGATACGTGGCCTTA
Ast2R		GTCGTTGCCAGAAAAGAAGC
Ast3F		TTGGAGAAAGGTCTGGATCG
Ast3R		GTCTCTCATGGTCCGGTTGT
Ast4F		ACCAGGATGCGCTGTCTAAT
Ast4R		GGCTGACCCACAGTGAGAAT

We used Canu (v1.6) for nanopore sequencing genome assembly [15]. De novo assembly of Sanger sequencing data was performed in DNAstar software [16]. Raw reads were remapped to the assembly using bwa (v0.7.12) and alignments were processed with SAMtools (v1.3) to obtain the consensus sequence [17, 18]. Amplicon sequences were used to fill the gaps depending on the overlapped regions with nanopore sequences. Represented strains of different HAstV genotypes were used for phylogenetic analysis. All sequences were aligned using MEGA (v7.0.21) [19]. Neighbor-joining trees were constructed using the Kimura two-parameter method, and reliability was assessed by bootstrapping with 1,000 resampling loops. Average Nucleotide Identities were calculated using the OrthoANIu tool [20]. SimPlot was used to visualize the relationships between the recombinant and its possible parents, with a window size of 200 nucleotides in length (nt) and a step size of 20 nt in the full-length HAstV genomes.

## Results and discussion

**Fig. 1:**
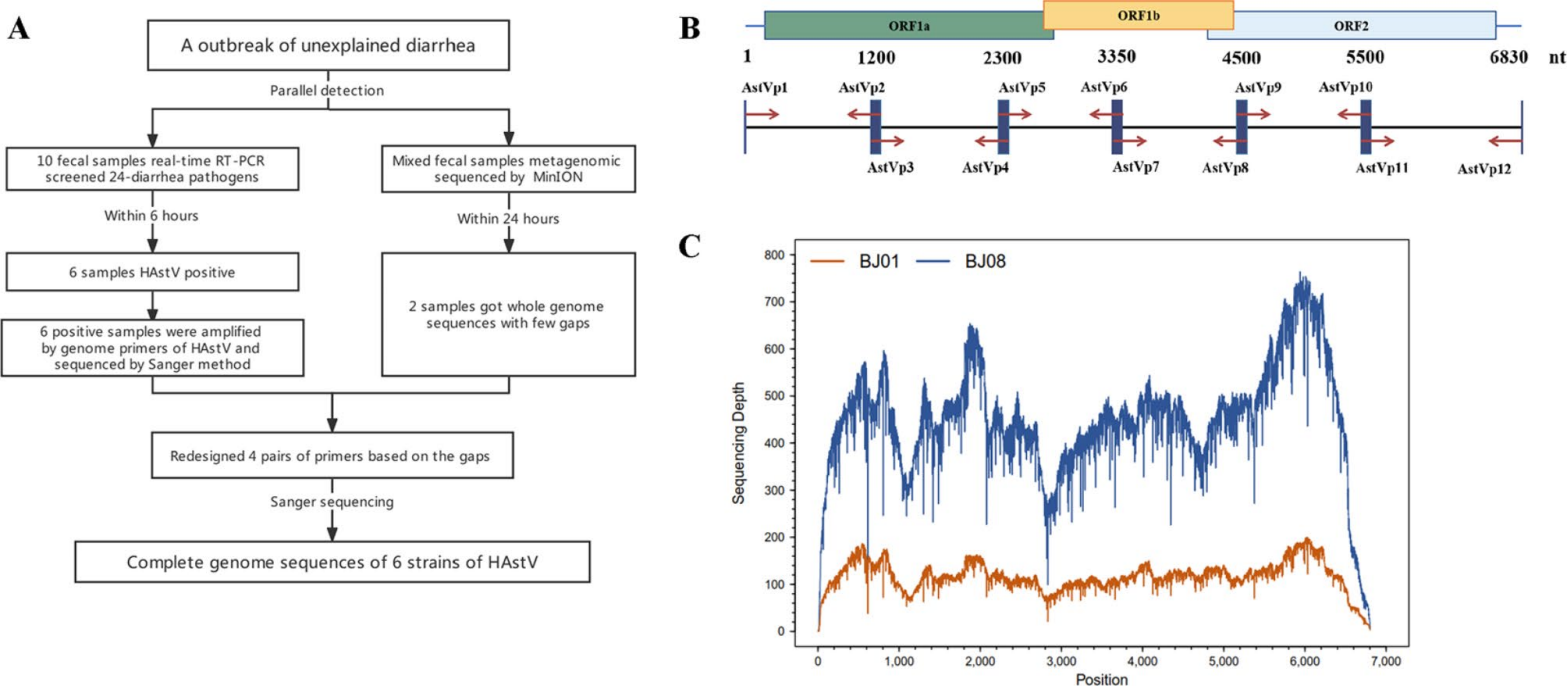
Overview of rapid pathogen detection and genome sequencing process (**A**). The locations of six pairs of primers on the HAstV genome (**B**), the sequencing depth of BJ01 and BJ08 (**C**)

The outbreak occurred in March 2019 in a training session in Beijing, China. Ten participants had unexplained diarrhea, and the duration of symptoms ranged from 1 to 3 days. A total of 10 (BJ01 to BJ10) fecal specimens were collected. We screened 10 samples for 24 enteric pathogens using multiplex RT-PCR, of which six (BJ01, BJ02, BJ04, BJ06, BJ08 and BJ09) were positive for HAstV while four samples were negative for all the 24 pathogens. Nanopore sequencing was performed on HSdtV-positive samples and two (BJ01 and BJ08) complete genome sequences were assembled, which generated 2421 and 592 reads, respectively. BLAST search against NCBI revealed that the outbreak strains belonged to serotype 5 and had the highest similarity with Yu/1-CHN(MG921619.1) [21]. BJ01 had a coverage of 99.89% and a depth of 117×, while BJ08 had a 100% coverage and a depth of 453×with Yu/1-CHN(MG921619.1) as the reference genome (Fig. 1C). To obtain the whole genome sequences of all HAstV-positive samples, 6 pairs of primers were used and the amplicons were sent for Sanger sequencing (Fig. 1B, Additional file 1: Fig. S1, S2). However, there were still some gaps that necessitated the design of specific primers to facilitate another round of amplification and Sanger sequencing (Additional file 1: Fig. S3). Finally, whole genomes of the six positive samples were obtained by combining nanopore sequencing and Sanger sequencing data (Fig. 1A).

**Fig. 2:**
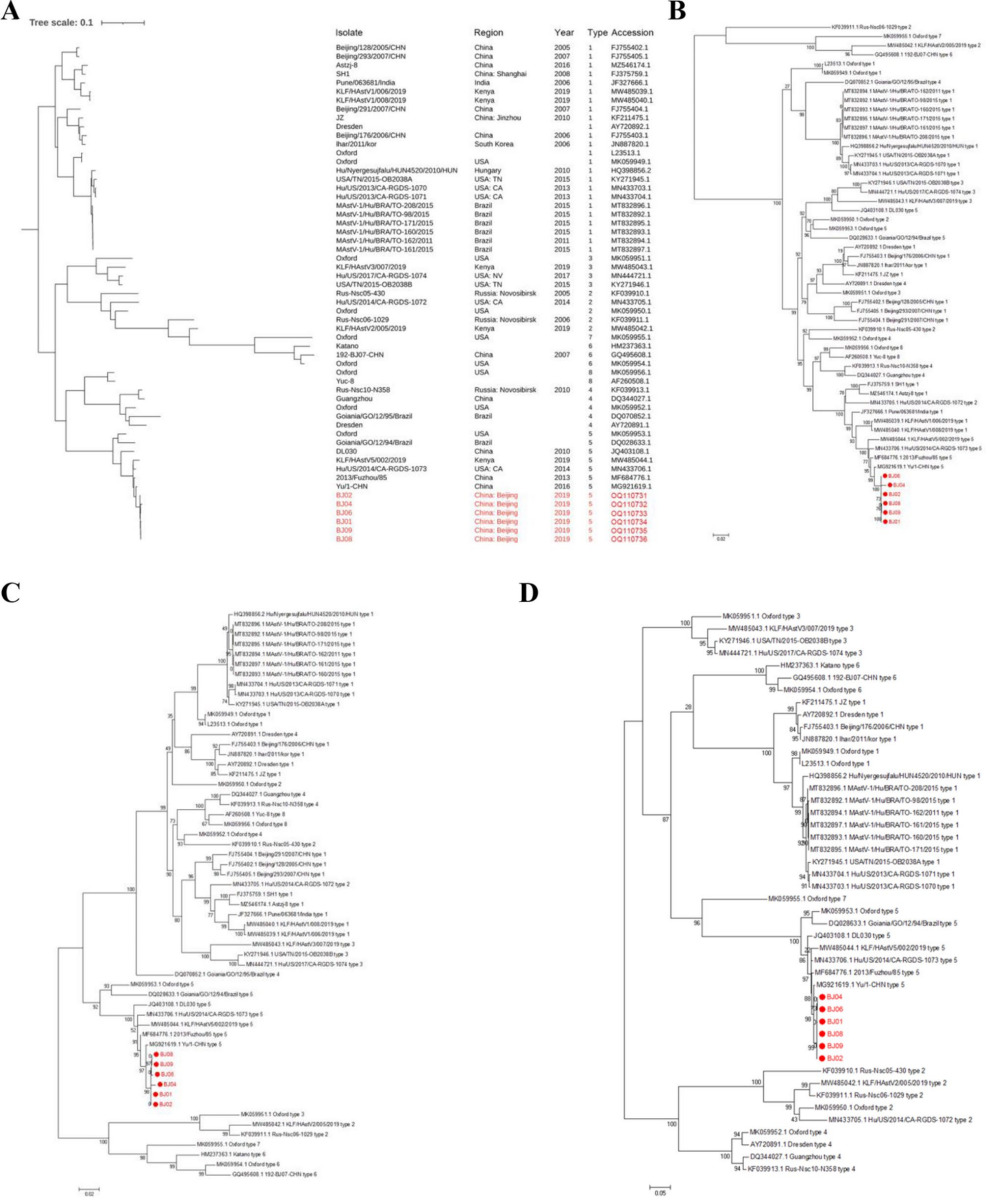
Phylogenetic analyses of the complete genomes (**A**), ORF1a(**B**), ORF1b(**C**) and ORF2(**D**) of 51 represented HAstV strains and the outbreak strains

Phylogenetic analysis was performed using all the 51 complete genomes of HAstV strains from the Genebank and the outbreak strains (Fig. 2). The tree showed that the six strains formed a distinct phylogenetic group and were closely related to domestic strains Yu/1-CHN(MG921619.1) and 2013/Fuzhou/85(MF684776.1). Phylogenetic trees of the three ORFs revealed similar relationships between the outbreak and other strains, while distances between the ORF1a and ORF1b segments of BJ04 and the other outbreak strains were longer than distances between the ORF2 segments.

**Fig. 3:**
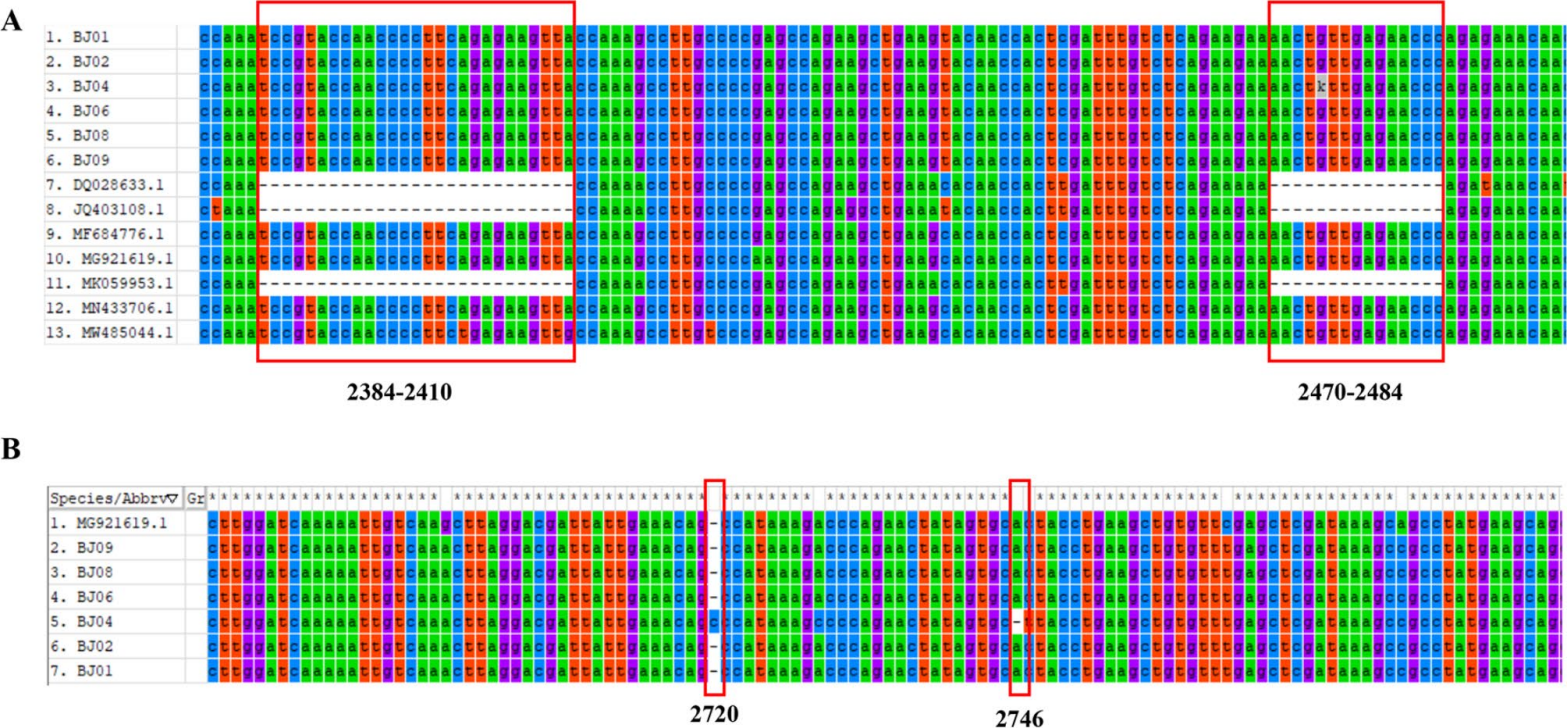
Comparative analysis of whole genome sequences among the six isolates(**A**), seven closest strains(**B**) and 13 HAstV-5 strains. Sequences of genes were aligned and visualized using MEGA7

Pairwise alignments revealed low-level nucleotide variations within the six strains. Whole genome sequence identities varied from 99.38 to 100%. All the six strains have acquired the 27-bp and 15-bp insertions in ORF1a compared with the early serotype 5 strains (Fig. 3A). Most variations of the outbreak strains were single nucleotide polymorphisms primarily located in ORF1a. A single-base insertion and a deletion were observed in BJ04 compared with other strains, resulting in eight amino acid changes in ORF1a (Fig. 3B).

**Fig. 4:**
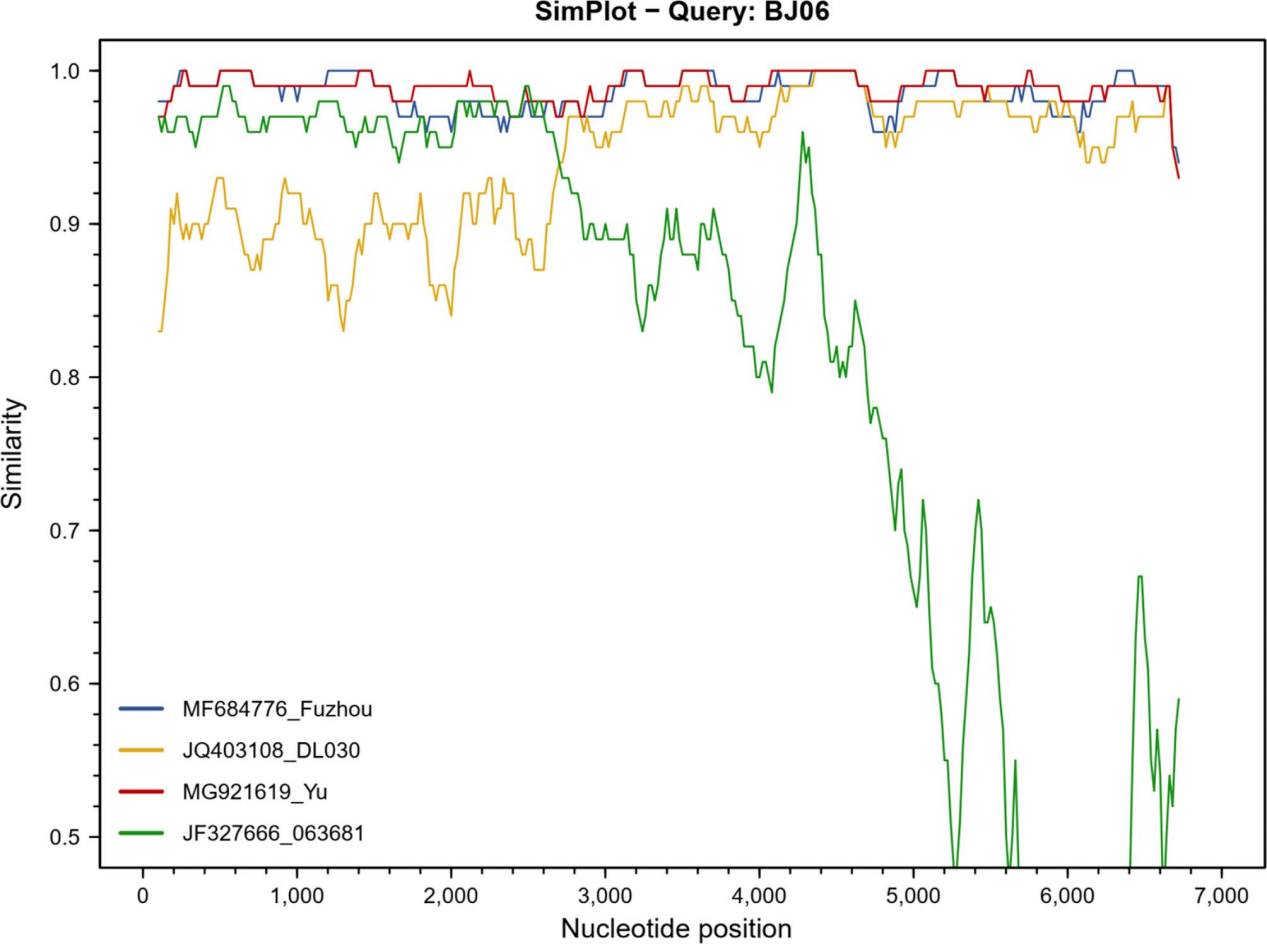
Homologous analysis of the whole genome of the BJ06 strain analyzed by Simplot software

Homologous comparison analysis using Simplot was performed to determine potential evolutionary origins. The BJ06 strain was used for comparison with 2013/Fuzhou/85 (MF684776.1), Yu/1-CHN (MG921619.1), DL030 (JQ403108.1) and Pune/063681/India (JF327666.1) (Fig. 4). ANI analysis showed that the recombination breakpoint of BJ06 was located at 2741 bp in the overlap region of ORF1a and ORF1b, which was the same recombination site of 2013/Fuzhou/85(MF684776.1) and Yu/1-CHN(MG921619.1). The distribution of ANI was consistent with homologous and phylogenetic analyses. The three ORFs of BJ06 were highly similar to those of 2013/Fuzhou/85 and Yu/1-CHN, with similarities of 89.09%, 97.22% and 95.58% to those of DL030.

HAstV have traditionally been regarded as uncommon causes of gastroenteritis, and are most often associated with mild disease, and may therefore be under-diagnosed. The detection rates of HAstV in acute gastroenteritis cases were previously reported at 3.0% in Guangzhou, 5.22% in Shanghai, 2.6% in Thailand, 2.8% in Russia and 5.0% in Germany, lower than the worldwide mean incidence of 11.0% [22–27]. The low detection rate might be explained by differences in sample size, geographic location, and detection methods [28], all of which have confounded the obtainment of the whole genome of HAstV.

To facilitate definitive diagnosis and accurate tracing, we used real-time PCR and nanopore sequencing in parallel for rapid detection. Six samples were positive for HAstV while 4 samples yielded no enteric pathogens. These 4 cases were attributed to food intolerance. However, only two complete genomes were obtained directly from HAstV-positive samples. Designed primers and Sanger sequencing enabled the filling of gaps and facilitated detailed genomic analysis during the outbreak.

Homologous recombination is a vital force driving evolution and contributes substantially to the genetic diversity of HAstV. Our outbreak strains had almost identical genetic variations and were similar to 2013/Fuzhou/85 and Yu/1-CHN, indicating that the outbreak strains had been circulating in China. Phylogenetic analysis showed that HAstV-5 comprised two groups, and that domestic strains had multiple short fragment insertions compared with foreign strains. Previously reported recombination breakpoints were identified within an upstream site of the ORF1a/ORF1b overlap region, then were frequent within ORF2, and were also located within ORF1a and ORF1b [29–33]. Our analysis reveals that the outbreak strains may have originated from recombination of DL030 and Pune/063681/India.

In summary, the parallel use of RT-PCR and sequencing enhances rapid diagnosis and the acquisition of whole genome sequences of HAstV. The whole genomes of the outbreak strains further demonstrate genetic diversity and recombination of HAstV-5. Nevertheless, long-term monitoring data are needed to determine the epidemiology of this circulating genotype. The combination of nanopore and Sanger sequencing unveiled genomic variation and recombination, and may provide crucial insights into the evolution of HAstV.

### Electronic supplementary material

Below is the link to the electronic supplementary material.

Supplementary Material 1

## Data Availability

The genomes of the human astrovirus isolates BJ01, BJ02, BJ04, BJ06, BJ08 and BJ09 have been deposited onto NCBI GenBank under accession numbers OQ110731 to OQ110736, respectively. The nanopore sequencing data of BJ01 and BJ09 have been uploaded onto SRA under accessions SRR22840003 and SRR22840002, respectively.
