# Supplementary material for: Characterisation of human astrovirus in a diarrhoea outbreak using nanopore and Sanger sequencing protocols
Source: Virol J. 2023 Nov 14;20:263. doi: 10.1186/s12985-023-02224-7 (PMC10647158; doi:10.1186/s12985-023-02224-7)

**Supplementary Information**

Additional file1 Fig. S1 The Gel plots of whole genome amplification products of Human astrovirus virus of BJ01, BJ02, BJ04, BJ06, respectively.


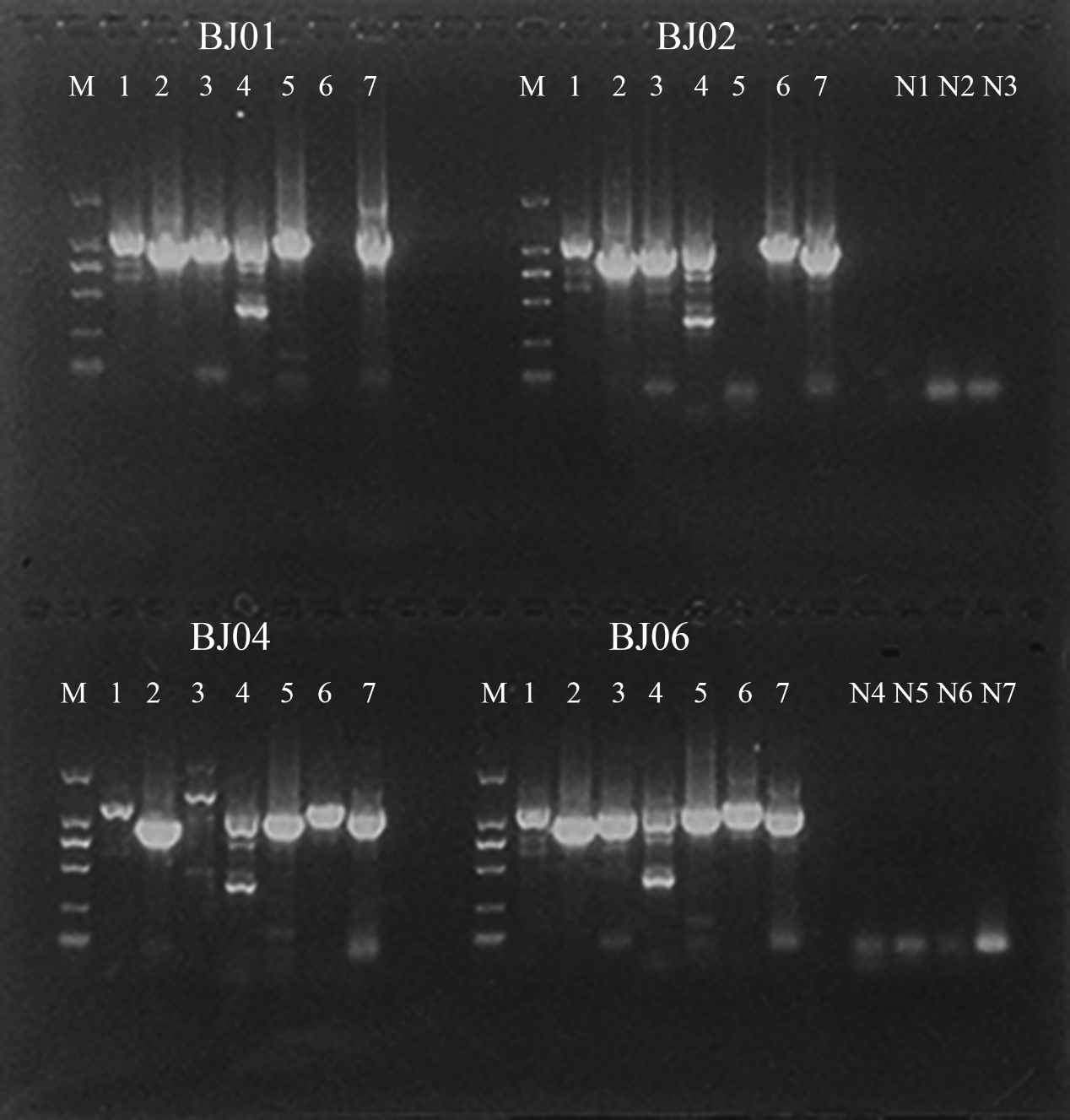


Additional file1 Fig. S2 The Gel plots of whole genome amplification products of Human astrovirus virus of BJ03, BJ05, BJ07, BJ08, BJ09, BJ010, respectively.


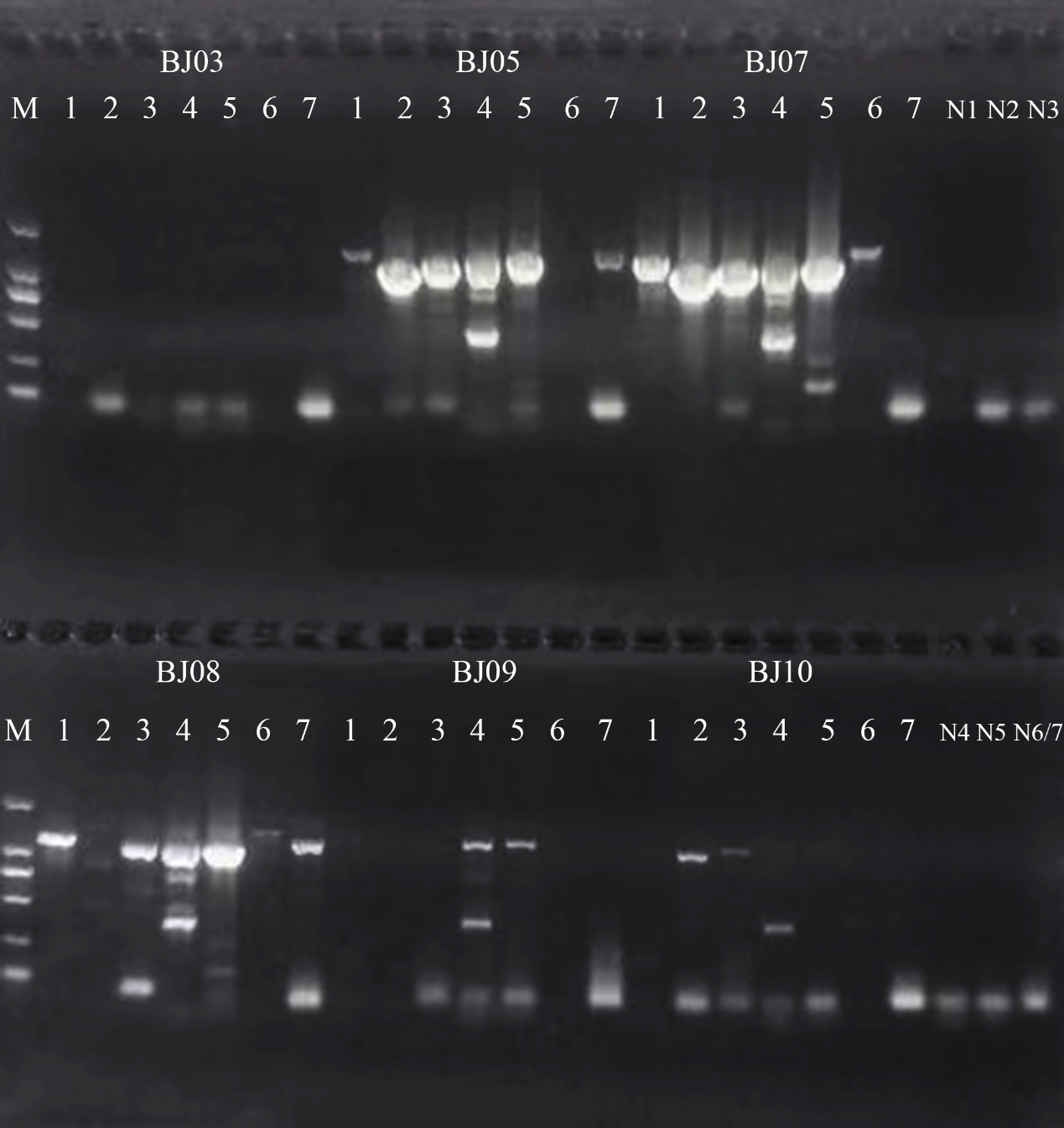


Additional file1 Fig. S3 The Gel plot of the amplification products for the gaps generated in sequencing.


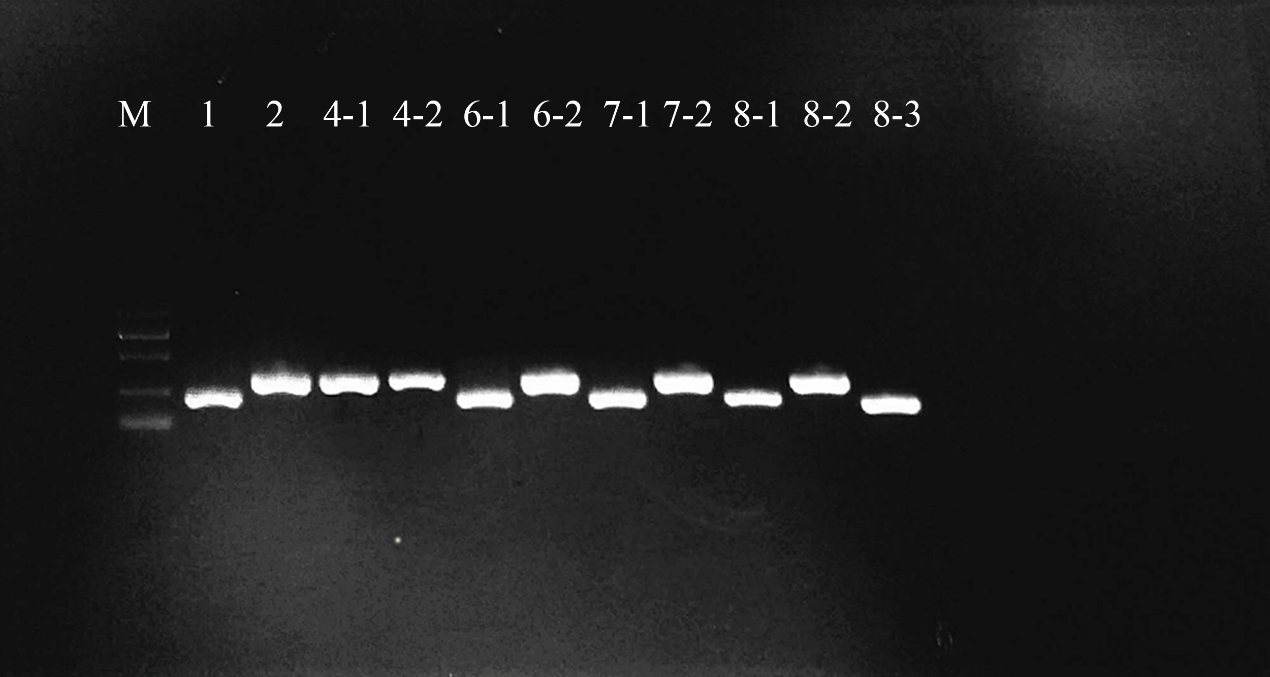

Supplement: Supplementary file 1 — Supplementary Material 1 [file 12985_2023_2224_MOESM1_ESM.docx]
